# Development of a predictive model using key ultrasound characteristics to distinguish follicular thyroid neoplasms from nodular goiter

**DOI:** 10.12669/pjms.42.6.14216

**Published:** 2026-06

**Authors:** Xiang Wei, Chenwei Wang, Zhaoyang Wu, Sufang Zhang

**Affiliations:** 1 Xiang Wei, Department of Medical Ultrasound, Department of Orthopedics, Fujian Orthopedics Research Institute, National Regional Medical Center, Binhai Campus, The First Affiliated Hospital, Fujian Medical University, Fuzhou 350005, China; 2 Chenwei Wang, Department of Medical Ultrasound, Department of Orthopedics, Fujian Orthopedics Research Institute, National Regional Medical Center, Binhai Campus, The First Affiliated Hospital, Fujian Medical University, Fuzhou 350005, China; 3 Zhaoyang Wu, Department of Orthopedics, Fujian Orthopedics Research Institute, National Regional Medical Center, Binhai Campus, The First Affiliated Hospital, Fujian Medical University, Fuzhou 350005, China; 4 Sufang Zhang, Department of Medical Ultrasound, National Regional Medical Center, Binhai Campus, The First Affiliated Hospital, Fujian Medical University, Fuzhou 350005, China

**Keywords:** Follicular thyroid neoplasm, Nodular goiter, Nomogram

## Abstract

**Objective:**

To develop a non-invasive diagnostic model by systematically quantifying and combining key ultrasound imaging characteristics for preoperative distinction.

**Methodology:**

We retrospectively analyzed 588 thyroid nodule cases (196 NG, 392 FTN) diagnosed between January 2012 to December 2024 at The First Affiliated Hospital, China. Variables identified through LASSO regression were subjected to multivariate logistic regression, and the resulting predictors were utilized to establish a nomogram. Cross-validation was performed to determine the optimal parameter, specifically the minimum λ (λmin). Model performance was rigorously evaluated using C-index, ROC curves, calibration curves, and decision curve analysis. Ultrasound image acquisition and interpretation were conducted in a blinded manner to minimize potential observer bias. Two-tailed P-values were used, with statistical significance set at P < 0.05.

**Results:**

The model demonstrated robust diagnostic performance with an AUC of 0.832 and excellent calibration. The three significant predictors were: single nodule (OR=3.662), presence of peripheral halo (OR=5.338), and rich blood supply (OR=6.075). Decision curve analysis confirmed substantial net benefits across threshold probabilities of 20-89%.

**Conclusion:**

This study establishes a clinically practical diagnostic model that effectively integrates and quantifies ultrasound features to differentiate FTN from NG preoperatively. The tool provides a valuable adjunct to conventional assessment, potentially reducing diagnostic errors and preventing unnecessary treatments for thyroid nodules.

***Abbreviations:* CI:** Confidence interval, **DCA:** Decision curve analysis, **FTN:** Follicular thyroid neoplasm, **LASSO:** Least Absolute Shrinkage and Selection Operator, **NG:** Nodular goiter.

## INTRODUCTION

Due to the widespread adoption of thyroid ultrasound screening, thyroid nodules are very prevalent with a detection rate ranging from 19% to 67%.^1^-^3^ While most detected nodules are benign (e.g., nodular goiter, NG), accurate identification of potentially malignant nodules (e.g., follicular thyroid neoplasm, FTN) is crucial to prevent overtreatment. The diagnosis of follicular thyroid carcinoma relies on the identification of ultrasonographic features indicating capsular or vascular invasion^4^, which requires surgical intervention. In contrast, NG typically presents as multinodular hyperplasia only requiring regular follow-up unless significant compressive symptoms develop.

Pathologically, FTN is a monoclonal neoplastic proliferation, while NG is formed by the continuous proliferation and involution of thyroid follicular cells and is non-neoplastic, encompassing both monoclonal and polyclonal proliferation.^5^-^6^ In conventional ultrasound examinations, follicular neoplasms often present as nodules with regular shape, clear margin, rich blood supply, and possible cystic changes. These features lack the characteristic sonographic signs of papillary carcinoma, such as microcalcifications, aspect ratio > 1, or markedly hypoechoic. This makes it difficult to distinguish FTN from NG on ultrasound images. Moreover, the Bethesda System for reporting thyroid cytopathology (categories III and IV) exhibits considerable interobserver variability and cannot assess capsular or vascular invasion, thus failing to provide an objective and reliable distinction between benign, indeterminate, and malignant nodules.^7^ Therefore, the preoperative differentiation between NG and FTN remains a considerable diagnostic challenge.

In response to the challenges in distinguishing between follicular thyroid nodules (FTNs) and nodular goiter (NG) using conventional ultrasound imaging and the Bethesda system, this study integrates and quantifies ultrasound imaging features to construct a visual model based on multimodal clinical and ultrasound data. This approach aims to improve the diagnostic accuracy of FTNs by clinicians and to avoid overtreatment of thyroid nodules.

## METHODOLOGY

This study included 625 patients with thyroid nodules who visited the First Affiliated Hospital of Fujian Medical University between January 2012 and December 2024, encompassing both NG and FTN pathological types.

### Inclusion criteria:

- Patients underwent total or partial thyroidectomy with postoperative pathological confirmation of NG or FTN.
- Ultrasound images of good quality with clear, multi-planar documentation of nodule size, margins, echogenicity, and blood supply.

### Exclusion criteria:

**Supplement Fig.S1:**
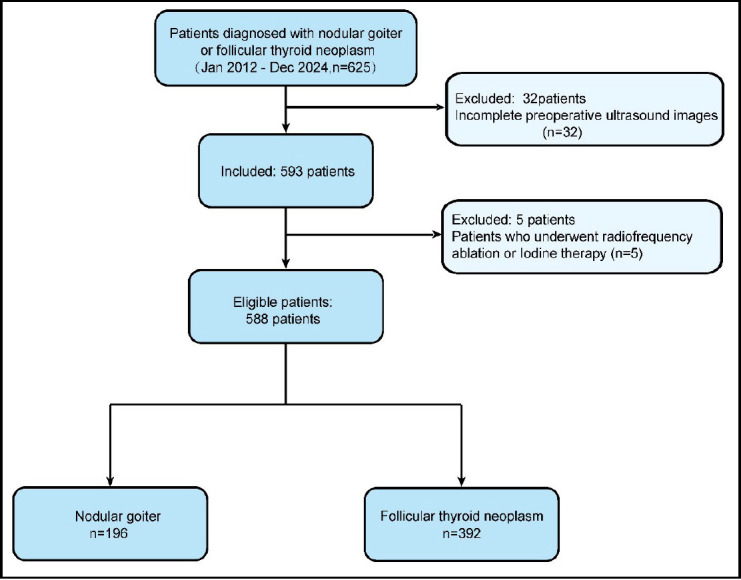
Flow diagram: After excluding ineligible data, this study ultimately included 588 patients with NG (n=196) and FTN (n=392).

- Incomplete preoperative ultrasound images, such as those with only gray-scale ultrasound without color Doppler flow imaging.
- Patients who underwent radiofrequency ablation or I^131^ therapy. After excluding ineligible data, this study ultimately included 588 patients with NG (n=196) and FTN (n=392). (Supplement Fig.S1)

### Ethical approval:

This study was approved by the Ethics Committee of the First Affiliated Hospital of Fujian Medical University. (No. 2015-084-2; dated January 13, 2022; valid until: April 1, 2025).

### Clinical information and ultrasound characteristics:

**Fig.1:**
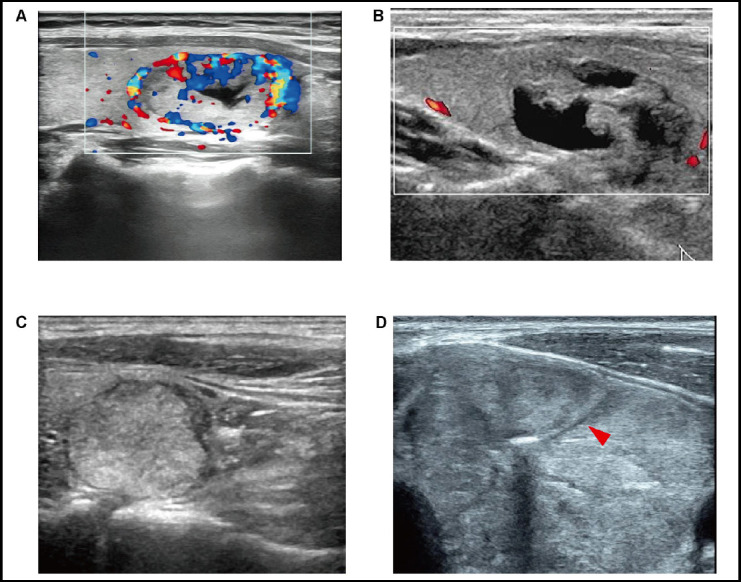
FTN and NG Ultrasound Images: A. Solitary thyroid adenoma with rich blood supply. B. Nodular goiter without peripheral halo, with relatively scarce blood supply. C. Thyroid follicular carcinoma with unevenly thickened peripheral halo. D. Thyroid follicular carcinoma showing the “separated nodule sign” (Red arrow).

This study employed a high-frequency color Doppler ultrasound diagnostic system with a linear array high-frequency probe operating at 12-14 MHz. Ultrasound examinations were performed by a senior physician with over five years of experience, who adjusted gain, depth, dynamic range, and focus to achieve optimal imaging quality and captured multi-plane images. Findings were confirmed by two senior physicians each with over five years of experience. Included indicators comprised age, gender, nodule location (left, right), number (single, multiple), size (maximum diameter), nodular aspect ratio, echogenicity (hypoechoic, isoechoic, hyperechoic), calcification (present, absent), composition (solid, mixed), and blood supply (scarce, rich) (Fig.1A, Fig.1B), peripheral halo (present, absent) (Fig.1C), and “separated nodule sign” (present, absent) (Fig.1D). The “separated nodule sign” refers to linear or band-like hypoechoic septa appearing within thyroid nodules on ultrasound images, dividing the nodule into several smaller segments.

### Model development:

This study employed LASSO regression analysis to screen clinical and ultrasound imaging features, selecting λ_min_ (the parameter yielding the smallest mean error) as the optimal parameter through cross-validation. The primary variables filtered by LASSO regression were incorporated into a multivariate logistic regression analysis. Statistically significant clinical and ultrasound variables obtained from the logistic regression were used to construct a diagnostic model, with a nomogram developed to visualize the model.

### Model validation and Evaluation:

This study evaluated model reliability by plotting ROC curves and calculating the AUC along with its 95% confidence interval. Calibration curves were used to assess the model’s predictive performance relative to actual outcomes. DCA was employed to evaluate the clinical net benefit of the model at different threshold probabilities. Net benefit was defined as the proportion of true positives minus the proportion of false positives.^8^,^9^ Internal validation of the constructed predictive model was performed using bootstrap resampling (repeated 1000 times) to assess model stability and generalization capability.

### Statistical analysis:

This study employed statistical analysis using R software (version 4.5.0). The “glmnet” package was utilized for LASSO regression analysis to screen variables among clinical and ultrasound imaging features, that is, to include ultrasound and clinical variables with non-zero coefficients. The “rms” package was employed for multivariate logistic regression analysis, plotting nomogram, calculating the C-index, and performing calibration curve analysis. Multivariate logistic regression analysis was used to establish a diagnostic model for FTN. Nomogram for visualizing FTN scores in the model. The C-index and calibration curve analysis assessed model accuracy.^10^ Additionally, the “ROCR” package was employed to generate ROC curves and assess the model’s diagnostic accuracy for FTN. The “rmda” package facilitated decision curve analysis to evaluate net benefits at different threshold probabilities. The “bootstrap” method was applied for model validation. Two-tailed P-values were used, with statistical significance set at P < 0.05.

## RESULTS

### Baseline information:

**Fig.2:**
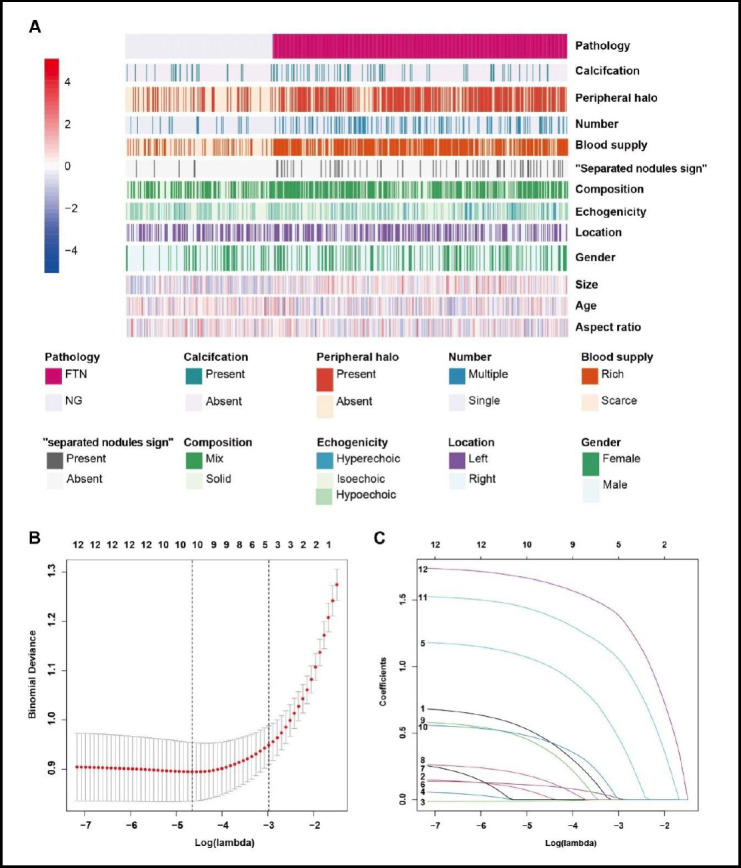
A. Clinical and ultrasound imaging data for 588 patients diagnosed with NG or FTN, where each row represents one variable and each vertical line represents one patient. Among them, thyroid nodules exhibiting ultrasound features such as calcification, peripheral halo, number, blood supply, and the “separated nodules sign” were significantly more common in FTN. B. Clinical features and Ultrasonic imaging features were screened by LASSO regression analysis. Two dashed lines correspond to λmin (0.010) and λ1SE (0.051) respectively. C. Each curve represents the change trajectory of the coefficients of each independent variable. The lower abscissa is log (λ), the ordinate is the value of the coefficients, and the upper abscissa is the number of variables (coefficients are not zero) in the model.

Based on prior research experience, this study incorporated 12 clinical and ultrasound characteristic indicators: age, gender, location, number, size, nodular aspect ratio, echogenicity, calcification, composition, blood supply, peripheral halo, and imaging characteristic. A total of 588 patients with thyroid nodules were ultimately included. Clinical and ultrasound examination indicators for each patient are presented as a heat map (Fig.2A). Among them, 196 patients were NG (49 males, 147 females), and 392 patients were FTN (133 males, 259 females) (Table-I).

**Table-I:** Clinical and ultrasonic feature of the thyroid nodules.

Variable	NG (n=196)	FTN (n=392)	Total (n=588)
Age (year, Mean±SD)	49.73	45.65	47.01±12.91
***Gender***			
Female	147 (75.00%)	259 (66.07%)	406 (69.05%)
Male	49 (25.00%)	133 (33.93%)	182 (30.95%)
Size (cm, Mean±SD)	2.60±1.67	3.71±1.75	3.33±1.80
Nodular aspect ratio (Mean±SD)	0.63±0.14	0.60±0.14	0.62±0.14
***Location***			
Left	97 (49.49%)	191 (48.72%)	288 (48.98%)
Right	99 (50.51%)	201 (51.28%)	300 (51.02%)
***Number***			
Single	18 (9.18%)	122 (31.12%)	140 (23.81%)
Multiple	178 (90.82%)	270 (68.88%)	448 (76.19%)
***Echogenicity***			
Hypoechoic	88 (44.90%)	155 (39.54%)	243 (41.33%)
Isoechoic	100 (51.02%)	197 (50.26%)	297 (50.51%)
Hyperechoic	8 (4.08%)	40 (10.20%)	48 (8.16%)
***Calcification***			
Absent	175 (89.29%)	329 (83.93%)	504 (85.71%)
Present	21 (10.71%)	63 (16.07%)	84 (14.29%)
***Composition***			
Mixed	93 (47.45%)	120 (30.61%)	213 (36.22%)
Solid	103 (52.55%)	272 (69.39%)	375 (63.78%)
***Peripheral halo***			
Absent	142 (72.45%)	113 (28.83%)	255 (43.37%)
Present	54 (27.55%)	279 (71.17%)	333 (56.63%)
***Blood supply***			
Scarce	139 (70.92%)	85 (21.68%)	224 (38.10%)
Rich	57 (29.08%)	307 (78.32%)	364 (61.90%)
***“Separated nodules sign”***			
Present	5 (2.55%)	69 (17.60%)	74 (12.59%)
Absent	191 (97.45%)	323 (82.40%)	514 (87.41%)

***Abbreviations:*** NG: nodular goiter; FTN: follicular thyroid neoplasm.

### Establishment of the diagnostic model:

LASSO regression analysis was performed on the 12 clinical and ultrasound feature variables included in the study (Fig.2B-2C) . After screening, 10 variables were selected: age, gender, size (maximum diameter), number, echogenicity, calcification, composition, blood supply, peripheral halo and “separated nodule sign”. Subsequently, these 10 variables were subjected to multivariate logistic regression analysis (Table-II), finally identifying three variables significantly associated with FTN occurrence: single nodule (OR = 3.662, 95% CI = 2.009-6.991, P≤0.001), hypoechoic halo (OR = 5.338, 95% CI = 3.232-8.980, P ≤ 0.001), and rich blood supply (OR = 6.075, 95% CI = 3.892-9.600, P ≤ 0.001).

**Table-II:** Multivariate logistic regression analysis.

Intercept and Variables	Prediction Model			
	β	Odds Ratio	95% CI	P value
Intercept	-1.859	0.156	0.082-0.285	<0.001
Age	-0.346	0.707	0.450-1.110	0.133
Gender	0.208	1.231	0.746-2.048	0.418
Number	1.298	3.662	2.009-6.991	<0.001
Size (cm)	0.028	1.028	0.633-1.664	0.901
***Echogenicity (Isoechoic)^a^***				
Hypoechoic	0.505	1.656	0.656-4.599	0.306
Hyperechoic	0.314	1.370	0.813-2.331	0.240
Calcification	0.571	1.771	0.919-3.511	0.094
Composition	0.445	1.561	0.960-2.540	0.072
Peripheral halo	1.675	5.338	3.232-8.980	<0.001
Blood supply	1.804	6.075	3.892-9.600	<0.001
“Separated nodules sign”	0.918	2.505	0.995-7.686	0.073

***Note:***

a the reference of hypoechoic and hyperechoic.

***Abbreviations:*** β, regression coefficient; CI, confidence interval.

**Fig.3:**
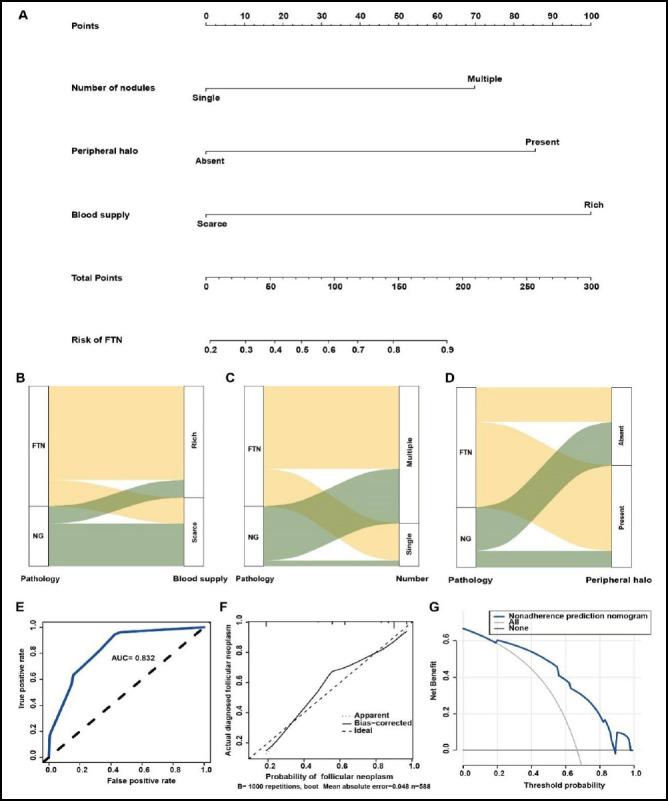
A. Diagnostic model for differentiating NG and FTN. This column chart comprises six rows, with rows 2–4 representing the ultrasound variables incorporated into the model. Each variable is assigned a score based on its regression coefficient, reflecting its weight within the model. For each patient, the sum of scores across all variables constitutes their total score. Mapping this total score onto the “risk prediction axis” allows for an intuitive reading of the patient’s probability of being diagnosed with FTN. B-D. Correspondence between NG or FTN and blood supply, number, and peripheral halo. FTN is mostly hypervascular, multiple, and with a hypoechoic halo; NG is mostly hypovascular, single, and without a hypoechoic halo. E. Accuracy evaluation of the FTN diagnostic model. ROC curve and AUC value (0.832) of the model. F. Calibration curve of the model. G. DCA Curve for the FTN Diagnostic Model. The horizontal axis represents threshold probability, while the vertical axis denotes net benefit. The gray solid line indicates the net benefit of the “treat all” strategy, the black solid line represents the net benefit of the “none treat” strategy, and the blue solid line shows the net benefit of using this model to guide treatment decisions.

Based on the above, three ultrasound variables were selected to construct a diagnostic model for FTN, visualized as a nomogram (Fig.3A). This model integrates multiple ultrasound features to distinguish FTN from NG. Meanwhile, Sankey diagrams provide an intuitive representation of the relationships between NG and FTN with thyroid nodule number, blood supply, and peripheral halo (Fig.3B-3D).

### Validation and evaluation of diagnostic model:

This study utilized ROC curves to evaluate the diagnostic performance of the model constructed from the above ultrasound variables for FTN. The AUC value was 0.832 (Fig.3E). The C-index further evaluated the model, yielding a C-index of 0.832 (95% CI = 0.799–0.865), indicating excellent diagnostic capability. Calibration curve analysis demonstrated that our model’s predictions closely matched actual outcomes, reflecting high accuracy (Fig.3F). Hosmer-Lemeshow test results: χ^2^ = 83.32, P = 0.67; a P-value greater than 0.05 indicates good model calibration.

Internal validation of the FTN diagnostic model was performed using the bootstrap method. After 1,000 bootstrap resamples, the validation group yielded a C-index of 0.834 (95% CI: 0.799, 0.866), further confirming the model’s high accuracy and practical value.

### Clinical application:

This study evaluated the clinical utility of the diagnostic model using DCA (Fig.3G). Results demonstrated that the model exhibited significant net benefit advantages within a threshold probability range of 20%–89%, outperforming both the “treat all” and “none treat” strategies. Within this interval, the model accurately identified patients with FTN while preventing overtreatment of low-risk NG patients.

## DISCUSSION

Our study successfully developed and validated a diagnostic model for distinguishing FTN, which integrates and quantifies multiple clinical and ultrasound imaging features to differentiate FTN from NG preoperatively. The diagnostic model demonstrated excellent discriminatory ability and provides a visual tool for individualized risk assessment, offering potential to support clinical decision-making and avoid unnecessary overtreatment.

Although there is partial overlap in the ultrasound features between FTN and NG, the presence of single nodule, hypoechoic halo, and rich blood supply significantly aids in the differential diagnosis of FTN. Patients exhibiting these characteristics demonstrate a markedly increased probability of FTN diagnosis. Our study indicates that rich blood supply carries the highest weight in the nomogram graph. This likely stems from FTN growth relying on pro-angiogenic factors such as vascular endothelial growth factor,^10^,^11^ whose stimulation induces extensive disorganized and tortuous neovascularization. In contrast, NG typically exhibits less pronounced vascular richness than FTN,^12^ with its increased blood supply being more compensatory and relatively orderly.

Independent risk factors for FTN in our study include single nodule. Research comparing thyroid cancer prevalence between single nodule (SN) and multinodular goiter (MNG) has shown a lower probability of thyroid cancer in MNG.^13^ Conversely, single thyroid nodule carry a higher risk of malignancy.^14^ However, some studies have also suggested that nodule number is unrelated to malignancy.^15^-^17^ Therefore, the current role of thyroid nodule number in thyroid differential diagnosis remains ambiguous. We included it as an auxiliary diagnostic indicator in our model but ultimately found this variable to be highly valuable for FTN diagnosis, serving as an independent risk factor.

The peripheral halo refers to the fibrous capsule surrounding the nodule. Our study indicates that the peripheral halo is a relatively important ultrasound feature for FTN. Previous studies suggest that thicker or unevenly thickened capsules are more likely to be associated with FTN,^18^ and the thickness of hypoechoic halo is positively correlated with the risk of FTN occurrence.^19^

However, due to the significant subjectivity involved in measuring halo thickness, we did not investigate halo thickness but instead used the presence or absence of a hypoechoic halo as an evaluation criterion. Calcification is often regarded as an indicator of malignant nodules,^20^ but it was not included in our model. The primary reasons are as follows: microcalcifications (psammoma bodies), which exhibit high diagnostic specificity, are a hallmark feature of thyroid papillary carcinoma^21^,^22^ rather than a common manifestation of FTN; FTN lacks papillary structures and rarely produces psammoma bodies; in NG, microcalcification-like echoes are predominantly caused by concentrated colloid rather than true calcification.^23^ Additionally, calcification was not statistically significant in the multivariate logistic regression analysis, hence its exclusion.

Although the “separated nodule sign” (*P*=0.078) was excluded in the multivariate logistic regression analysis in this study, it may still hold some diagnostic significance. The “separated nodule sign” is defined as a large oval nodule divided into several smaller nodules by hypoechoic septa. Some studies refer to this as the “nodule-in-nodule”, 24 while others refer to it as the “cluster of grapes sign”,^25^ These terms describe features similar to the “separated nodule sign,” and in their studies, this ultrasound feature suggested a potential for malignancy. Furthermore, the “separated nodule sign” is easily recognizable by clinician, making it a useful indicator for distinguishing nodular goiter from follicular thyroid tumors.

### Strength of this study:

The principal strength of this study lies in its systematic integration of multiple quantifiable ultrasound features into a visual nomogram, effectively bridging the diagnostic gap between conventional imaging and histopathology for differentiating FTN from NG. Unlike previous studies that relied on subjective assessment, our model utilizes LASSO regression and multivariate logistic analysis to identify three independent predictors—single nodule, peripheral halo, and rich blood supply—thereby minimizing observer bias and providing a standardized, numerical risk assessment tool. The robust internal validation (AUC=0.832, Bootstrap C-index=0.834) confirms its reliability and potential to reduce unnecessary surgical interventions for indeterminate nodules.

### Limitations:

First, as a single-center retrospective study, our findings are susceptible to inherent biases, including selection and information bias. The generalizability of our results may be constrained by the specific demographic characteristics, surgical indications, and institutional protocols of our center, potentially limiting the applicability of our conclusions to broader populations. Second, regarding the sample size disparity between NG and FTN, although the natural incidence of NG is higher, the majority of NG cases are asymptomatic or managed conservatively without surgery. Consequently, the surgically treated NG cohort in our database was significantly smaller than the FTN cohort. This sampling skew may introduce spectrum bias and does not fully reflect the true epidemiological distribution of thyroid nodules.

## CONCLUSION

In recent years, artificial intelligence has progressively played a significant role in the field of ultrasound. This study combines and quantifies ultrasound imaging features, converting various clinical and ultrasound characteristics into scores for different variables. These scores are then converted into specific probability values, thereby reducing subjective bias and aiding in the differentiation between FTN and NG. This approach holds promise for lowering the misdiagnosis rate of thyroid nodules and consequently reducing unnecessary surgical interventions.

### Recommendations:

Therefore, future large-scale, multi-center prospective studies are essential to validate our findings, establish standardized diagnostic criteria, and minimize the impact of single-institution practice patterns.

### Authors’ contributions:

**XW & CWW:** Software, validation, formal analysis, investigation, resources, data curation, writing-original draft preparation.

**SFZ & ZYW:** Conceptualization, methodology, writing-review and editing, visualization, supervision, project administration, funding acquisition.

All authors have read and approved the final version of the manuscript.

**SFZ** is responsible for the overall integrity of the study.
